# The Importance of Lived Experience: A Scoping Review on the Value of Patient and Public Involvement in Health Research

**DOI:** 10.1111/hex.70205

**Published:** 2025-03-26

**Authors:** Heather Mah, Ruth Dobson, Alison Thomson

**Affiliations:** ^1^ Centre for Preventive Neurology, Wolfson Institute of Population Health Queen Mary University of London London UK

**Keywords:** benefit, health research, patient and public involvement, scoping review, social capital, value

## Abstract

**Background:**

Recently, patient and public involvement (PPI) in research has gained significant attention, particularly within the United Kingdom. Although there has been a growing focus on the impact of PPI on research outcomes, there remains an important gap in understanding its effects on the individuals involved and the value they gain from their participation.

**Objective:**

This scoping review aims to critically examine how PPI benefits both people with lived experience and researchers, shedding light on the value of their involvement in shaping research.

**Methods:**

We searched MEDLINE, PsycINFO, EMBASE, Cochrane and Web of Science for full‐text articles published in English after 1996. Grey literature searches reviewed policies from international research funders and patient organisations. Two reviewers independently carried out the abstract, title and full‐text article screening stages. Data abstraction was performed by one reviewer and verified by a second reviewer. Thematic analysis synthesised the findings.

**Results:**

After searching 3024 citations, 107 published articles and nine unpublished resources were included in the review. Most of the studies were conducted in the United Kingdom in the last 10 years. Thematic analysis of the value of PPI revealed four main themes: (1) value from contributing to research, (2) importance of relationships, (3) attitudes and support for PPI and (4) emotional labour of involvement.

**Discussion:**

This scoping review reveals the significant contributions alongside systemic challenges of PPI in health research. Being valued was framed as an impact of PPI to both PPI advisors and researchers. It emphasises the importance of social capital in developing relationships between researchers and people with lived experience yet highlights barriers that can hinder effective collaboration. This can lead to experiential knowledge being undervalued as a crucial perspective to inform research. Despite people being chosen to take part on account of their knowledge, skills and lived experience, these resources were not always used to their full potential due to researchers' expectations and restrictive research and institutional processes. The review calls for coordinated efforts to improve how PPI is valued and practised beyond a process or method to ensure PPI is done thoughtfully and effectively.

**Acronyms**
GRIPP2: Guidance for Reporting Involvement of Patients and the Public
NIHR: National Institute for Health and Care Research
PPI: patient and public involvement
PRISMA‐ScR: Preferred Reporting Items for Systematic reviews and Meta‐Analyses extension for Scoping Reviews

## 1 Introduction

Patient and public involvement (PPI) in health research has gained significant attention for its potential to enhance the relevance, quality and impact of research outcomes. PPI aims to democratise the research process by involving patients, carers and the public across the research process, including priority setting, study design, data collection, analysis and dissemination. People use their experiences to improve planning and delivering research, rather than being a research subject in a study [1].

Although the ‘impact’ of PPI, the measurable changes to research outcomes or processes, has been a dominant focus of evaluation [2, 3, 4, 5, 7], it does not fully capture the nuanced significance of involving patients and the public. Alternatively, ‘value’ shifts the focus from measurable outcomes to understanding what is meaningful to those involved. This links with its democratic roots where involving people who are affected by the outcomes of research, by having a say in how it is conducted, is not just a method for better research outcomes, but a fundamental right [7]. Value considers whether PPI can help develop trust, and how it enhances (or hinders) the research experience for those involved. Despite its potential, the concept of value remains underexplored in PPI.

To better understand the value of PPI, exploring its theoretical underpinnings, particularly the concept of social capital, can provide a deeper understanding of its relational and social benefits. Social capital is the networks, trust, reciprocity and social norms from interactions within groups, enhancing collaboration and shared knowledge [8]. Through PPI, people can develop relationships with researchers and peers, strengthening collaboration and fostering a shared purpose. This perspective shifts the focus from outcomes, such as improved recruitment, to relational dynamics that can enable collaboration. People's perceptions of value from being heard, respected and contributing to the greater good [2, 9, 10] align with the broader concept of social capital driven by mutual benefit.

Despite the growing recognition of the benefits of PPI in health research, there remains a need for a comprehensive synthesis of existing literature to better understand the multifaceted value that individuals get from participating in PPI activities. We seek to better understand how the concept of value has been considered in PPI, particularly the ways it can foster the development of key relational factors such as trust and reciprocity among individuals and groups engaged in health research. This is crucial to ensure that PPI is not only measured for what it achieves but how it is experienced and perceived by everyone involved.

## 2 Methods

This review was conducted using the methodological framework proposed by Arksey and O'Malley [11] which involves six stages: ‘1) Identify the research question, 2) Identify the relevant studies, 3) Study selection, 4) Charting the data, 5) Collating, summarise and reporting the results and 6) Consultation (optional)’ (p. 22). In addition, we incorporated recommendations from Levac et al. [12], who emphasised the importance of clarifying the research purpose and using an iterative approach in data selection. The review was designed to follow the Preferred Reporting Items for Systematic reviews and Meta‐Analyses extension for Scoping Reviews (PRISMA‐ScR) [13] (Additional File S1). The protocol for this review was published online in April 2022 (https://osf.io/d2zhc/).

### 2.1 Patient Involvement

Before this review, we held an online focus group with seven patient advisory group members (four women, three men) living with long‐term health conditions, representing different ages, ethnicities and educational backgrounds. Specific demographics are not provided to maintain group members' privacy and anonymity. Their feedback directly shaped the scoping review questions. They highlighted the value of contributing to PPI through giving back, staying informed and connecting with peers, which informed our first question on participants' personal gains and motivations. Their discussions on how they were invited into the group showed the importance of exploring the skills required, shaping our second question. Concerns about delayed payments, lack of recognition and poor communication guided the third question on improving systems for fair treatment and effective communication. This feedback provided important context for addressing both the benefits and challenges of PPI.

### 2.2 Objectives

This scoping review aimed to explore the value PPI advisors and researchers derived from participating in PPI in health research. Central to this exploration is understanding relational factors, such as trust and reciprocity, and how they contribute to effective PPI. These elements can foster environments where advisors feel valued, respected and able to contribute. Three specific questions identified for this review were the following: (1) What do people get from participating in PPI? (2) Which skills/knowledge do advisors require to contribute? (3) What processes and practices ensure the meaningful involvement of advisors in health research? By framing trust, reciprocity and cohesion as central to PPI, we aim to highlight how these relational aspects underpin the broader value of involvement.

### 2.3 Inclusion Criteria

Published and unpublished full‐text articles that explored how people with health conditions and researchers reported how they benefitted from PPI in health research through qualitative and mixed methods were included. The language was restricted to articles available in English. Full text was unavailable for 40 studies; after exploring all available access points, these were excluded from the final review to ensure data completeness and accuracy. We also excluded book chapters, reviews, editorials, opinion pieces, abstracts only and commentaries.

During the full‐text screening phase, although we had planned to exclude commentaries in this review, some commentaries were written or co‐authored by people with lived experience and provided useful information about their personal reflections and experiences of PPI. Therefore, these four articles were included [14, 15, 16, 17]. Based on findings from the initial search, we updated the inclusion criteria to include commentaries to provide broader perspectives on involvement.

### 2.4 Search Strategy

We conducted a keyword search in the following electronic databases: MEDLINE, PsycINFO, EMBASE, Cochrane and Web of Science. The search terms were developed with support from faculty liaison librarian with experience in conducting literature searches and based on previous PPI scoping reviews [18, 19, 20]. Keyword search terms were the following:

1. Value OR benefit OR compens* OR payment OR capital*.
2. ‘Patient involvement’ OR ‘patient engagement’ OR ‘public involvement’ OR ‘public engagement’ OR ‘patient and public involvement’.
3. Health*.
4. Research.
5. 1 + 2 + 3 + 4.

Articles were published after 1996 to 26 October 2021 when the search was conducted. We chose 1996 as this is when PPI became formalised in the United Kingdom. We updated the search using the same databases and search terms up to September 2024 to see how PPI literature had developed.

### 2.5 Grey Literature

To incorporate additional sources of information to the published journal articles, we also reviewed some grey literature on PPI. The grey literature search was conducted in October 2021 and September 2024 and included targeted online searches of international government, research funders, academic and charity organisation websites (Additional File S2). We included nine policies and guidance that were relevant to the review questions (Additional File S3). Incorporating these documents helped fill gaps in the academic literature, offering insights to understand how different healthcare systems, institutions and funders frame how they value advisors and their contributions in their guidance and policies.

### 2.6 Data Extraction and Synthesis

We used Covidence software [21] to manage the study selection between reviewers and data extraction. Two reviewers independently reviewed the titles and abstracts (Phase 1) against the eligibility criteria, discussed any conflicts and resolved by consensus. We then independently reviewed full texts of included studies (Phase 2) and again discussed any conflict and resolved by consensus. One reviewer extracted the data (Phase 3) from the chosen studies using an adaptation of the Guidance for Reporting Involvement of Patients and the Public (GRIPP2) long form, which provides guidance on reporting involvement in research studies [22]. A sample of these studies was checked by another reviewer to validate the extracted data and codes.

Other data extraction information included the following:

1. Publication details (author, year and country).
2. Health research setting.
3. Inclusion and exclusion criteria.
4. PPI keywords and definitions.
5. Value of PPI to people (e.g., financial, social and educational).
6. People being valued by research team (e.g., training, induction and feedback).
7. Skills required to do PPI (e.g., health/IT literacy, previous experience).
8. Trajectory of PPI involvement (e.g., moving on, transferring skills).

### 2.7 Collating and Summarising Data

We used Braun and Clarke's [23] approach for thematic analysis to identify themes. We used both deductive coding based on an a priori template of codes approach [24] developed from consultation with a patient advisory group, as well as used the data‐driven inductive coding approach to identify any new themes [25]. We uploaded extracted data into a Microsoft Excel spreadsheet to organise codes and look for common themes. We kept a codebook with code labels, description, source data and related themes.

## 3 Main Results

### 3.1 Description of Studies

**Figure 1:**
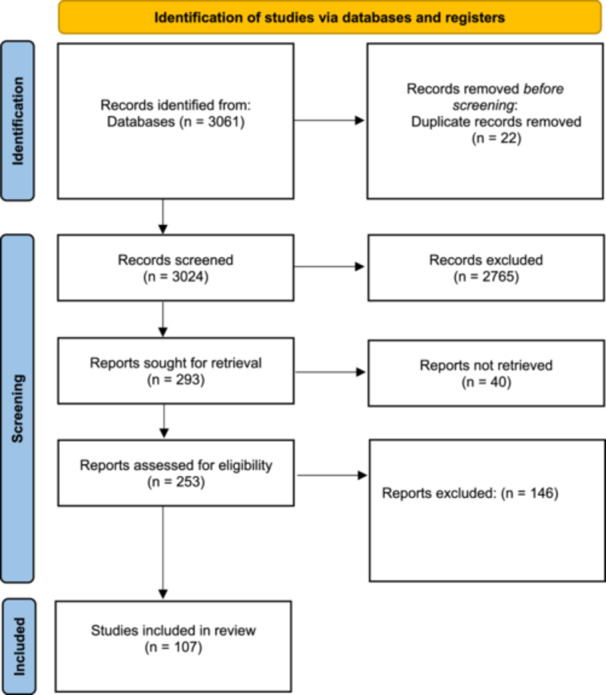
PRISMA Scoping Review flow chart [26].

The search yielded a total of 3024 articles, comprising 2077 published between 1996 and 2021, and 947 published from 2021 to 2024. After the removal of duplicates and abstract screening, we assessed 256 articles and included 107 published articles and nine grey literature resources (Figure 1). The earliest article included in this review was from 2007, from 2014 onwards the literature on the topic increased dramatically, demonstrating an expanding research focus on people's experiences of participating in PPI.

Articles included a range of health conditions as well as studies that involved multiple health conditions and some with general health conditions. However, the most common health conditions were cancer and mental health, which reflects findings that most published involvement literature is in these two areas [27]. Qualitative interviews were used in 55 studies, 23 used surveys or questionnaires, 19 were commentaries or personal reflections, nine conducted focus groups and five were evaluations.

Studies were conducted across 15 different countries, including 52 in the United Kingdom, 13 in Canada, six in the United States and the Netherlands and five in Sweden and Australia, reflecting the established PPI tradition in the United Kingdom [28].

### 3.2 Findings

This scoping review sought to understand the value PPI advisors and researchers got from participating in PPI, the skills and knowledge required and the process and practices that facilitate effective involvement. PPI advisors were often older, of White ethnicity, highly educated with professional backgrounds. In many published articles, people brought previous personal or PPI experience to the role [29, 30, 31, 32, 33, 34, 35, 36, 37, 38, 39, 40].

The key themes identified were (1) value from contributing to research, (2) importance of relationships, (3) attitudes and support for PPI and (4) emotional labour of involvement (Additional File S4). There were also instances where experiential knowledge was not considered equal to academic knowledge. Developing trusting relationships was essential for advisors to be treated as equal members of the research team. However, researchers found incorporating experiential knowledge into research challenging, which was affected by institutional research processes.

### 3.3 Grey Literature Results

Grey literature revealed that many organisations and funders lacked up‐to‐date, accessible information on PPI. Some web pages had not been updated in over a decade, and patient engagement frameworks were outdated, often without publication or review dates. Although organisations expressed a commitment to PPI, they provided lengthy, formal documents that were not user‐friendly and offered limited guidance on how people could get involved.

Grey literature resources conceptualised value in PPI in four ways: (1) values or principles of involvement, (2) PPI as adding value to research, (3) advisors feeling valued for their contributions and (4) researchers feel their work is of value [41, 42, 43, 44] (Henley 2020). Value was strongly related to the idea of meaningful involvement in research. Organisations offered principles to engage meaningfully with patients and the public. We have mapped these against the UK Standards for Public Involvement [45] which includes valuing contributions and building relationships in their principles and values of PPI. (Additional File S5). Notably, there was less of a focus on assessing impact. Grey literature also identified codeveloped tools to track and record how people contributed, what changes were made and why these changes matter [46, 47]. Such resources can help research teams to be transparent, have open discussions and reflect on experiences shared and their impact.

### 3.4 Theme 1: Value From Contributing to Research

There were different ways that PPI advisors expressed feeling valued. Some were through tangible aspects of involvement by gaining new skills and knowledge and accessing new opportunities. For example, they learned how to deliver research including data collection, data analysis, coding, co‐authoring papers, dissemination and presenting at conferences [33, 48, 49, 50, 51]. They also gained increased knowledge about their health condition, treatments and causes [36, 52, 53, 54, 55, 56]. Being involved in research gave advisors a sense of belonging and purpose, opportunities to meet others in similar circumstances and engage with researchers outside of a clinical setting. This was important for those who could no longer work due to their health or carer commitments [14, 33, 36, 37, 57, 58].

Payment for involvement acknowledged the economic value of advisors' time and experiences. There was an increased discussion on fair payments for involvement and non‐monetary alternatives [59, 60, 61]. Although receiving compensation made some advisors feel appreciated, others volunteered without payment. Payment rates varied, often going unreported and institutional processes sometimes delayed compensation, particularly for those lacking an email, bank account or receiving state benefits (Additional File S7). Some studies noted logistical challenges and limited funding for payments [62, 63]. However, alternative methods like salaried roles [64] and honorary contracts [51] were used. Additionally, discussions around fair compensation, including paying advisors more than researchers to address socioeconomic barriers, faced resistance from ethics boards insisting on equal pay [65]. In contrast, some studies successfully paid patient partners and researchers different rates [66].

Another way to recognise efforts to research is contributing to journal articles and being included as co‐authors [67]. Although advisors were listed as co‐authors in just over half of the articles, it was not always clear what their contributions were, as studies were written from the researchers' perspectives. In one article, lay observers were thanked for their contributions to the paper but not listed as co‐authors [34]. Only a few articles provided first‐person descriptive accounts of involvement from the perspective of patients and carers. One person with dementia wrote about their firsthand experiences of being a research network member [16]. In another study, lay researchers who cared for someone with dementia shared reflections of their involvement experiences from personal diaries [32]. Whereas intrinsically valuing individuals and their experiences depended on the extent they were included in research projects. It was important for advisors to be involved in discussions, listened to and receive regular feedback and communication [15, 33, 49, 68]. They wanted to feel their contributions not only were appreciated but also had the potential to shape research and make an impact [6, 33, 64]. In one study, advisors felt valued when they used their skills and experience to deliver training [17]. In another study, researchers attending involvement meetings and contributing to meeting agendas were important [49]. For others, they valued being provided with a mentor or buddy to support them [69, 70, 71].

However, experiential knowledge was not always valued or given ‘equal status’ [72] and instead was discounted for being non‐academic [58]. Clinicians could be dismissive of PPI, resulting in people feeling underused and not recognised for their skills [73]. One advisor felt the researchers talked over them, cut them off and expected advisors to agree with them [68]. People were considered problematic if they did not fit into a designated role [35] and felt undermined for not conforming to rigorous academic models [32].

### 3.5 Theme 2: Importance of Relationships

Several studies identified the need for nurturing and trusting relationships between researchers and PPI advisors for individuals to feel valued [17, 32, 33, 38, 39, 49, 74]. It was helpful to have informal opportunities to develop relationships and have space for discussion outside of formal meetings [75]. However, these opportunities had been reduced with meetings been held online with changes in working patterns because of Covid‐19 [76, 77, 78, 79, 80]. Online discussions and formalities can impact relationship building such as the ability to show empathy and read body language [81].

Relationship building was important to researchers who had strong motivations and commitment towards PPI. The development of strong relationships meant that PPI advisors felt they were valued and treated as equal members of the team [16, 34, 35, 49, 54, 82] and their contributions were recognised, taken seriously and appreciated [50, 56, 71, 83]. Studies identified the importance of developing trust and rapport between advisors and researchers required time [32, 54] which enabled people to feel comfortable to discuss sensitive topics [49]. One study held meetings in lay researchers' homes to create informal safe spaces outside of the research environment [32]. In some cases, relationships developed that lasted beyond the research project [81].

Strong relationships influenced whose contributions were included as many PPI advisors were selected or approached by someone on the research or clinical team. This was due to being previous research participants, already known to researchers, or chosen based on their experience or knowledge [17, 82]. Although some researchers acknowledged that PPI relationships differed from the doctor–patient relationship [56], others continued to consider PPI advisors as patients [33]. In one study, advisors experienced negative reactions from peers who felt that they got special treatment due to their relationship with their clinician [33].

### 3.6 Theme 3: Attitudes and Support for PPI

Broader organisational support and attitudes towards involvement impacted how PPI was considered and delivered. For researchers to have the capacity to embed involvement into their research depended on how invested their colleagues, managers, organisation and funders were in PPI. For example, by providing researchers with support, time and resources to plan and deliver activities and meetings [15, 49, 57, 71]. Sometimes when researchers tried to incorporate experiential knowledge meaningfully, they faced criticism from funders [84] and bureaucratic recruitment processes [34]. PPI advisors with previous work experience and education felt out of their depth in meetings [33] and found presenting their own issues during patient board meetings a struggle [56]. Only one study involved interpreters during a PPI meeting [83].

The extent to which lived experience was included in research depended on funding requirements and how motivated and committed researchers were to do PPI. In several studies, researchers were unfamiliar with PPI [56, 85] often had no previous experience facilitating PPI [39] and no formal training [6]. This meant that researchers had to learn new skills not currently taught in research on how to engage and support people to take part and listen effectively [63]. There were examples of researchers and institutions thinking that PPI counted negatively towards research, impacts deadlines and harms career prospects [35, 58, 73, 86].

Several articles involved advisors in qualitative evaluation studies assessing the effectiveness of the PPI they advised on as the intervention. At times researchers facilitating the PPI also conducted evaluation of their own projects [14, 29, 48, 70, 73]. This could limit advisors' ability to be critical and honest with researchers while creating a bias towards having positive results and due to positive motivation and belief in the benefits of PPI [49, 64].

### 3.7 Theme 4: Emotional Labour of Involvement

This theme highlights the emotional efforts that individuals invest in sharing personal, sometimes painful experiences, building trust and navigating vulnerability in PPI. Although in some cases, people felt valued for their contributions, the act of contributing also had emotional consequences. Certain aspects of PPI negatively impacted advisors' physical and mental health due to the demands of their roles. They also had to manage periods of ill health and emotional distress from discussing their health with others. A common experience from doing PPI was fatigue [33, 55], preparing for meetings was exhausting [85], the complexities and pressures of the work were overwhelming [49] and too much time commitment [63]. In some instances, advisors could no longer continue doing PPI due to physical health needs [6], carer responsibilities [87], family commitments [31] and personal reasons [56, 57]. The views of advisors no longer involved in PPI were not available or included in the research [56].

Sharing personal experiences was draining and emotionally challenging [49, 88]. Particularly when advisors had disabling and terminal conditions, like motor neuron disease, where they would be reminded of their health and worried that other members would die [55]. With spinal cord injury, advisors feared losing hope if there were negative research results and that it would confirm there is no cure [89]. Some advisors felt ‘put on the spot’ having to share personal experiences in different situations [58]. They also expressed concern about appearing confrontational and too pernickety [30]. However, sharing experiences also allowed advisors to reframe painful memories [90], deal with their diagnosis [72] and accept their disability [91].

## 4 Discussion

Given what is known about the impact of PPI on research outcomes, this scoping review sought to explore the literature on the value derived from involvement. Findings show the importance of involving people in research as a key principle, bringing value and reflecting people's own values rather than a process or method [92]. We also found that being valued was framed as an impact of PPI on both advisors and researchers. Doing PPI gave researchers reassurance that their work was of value and advisors a sense of belonging and that they were doing something useful.

These findings are interesting because they play into the concept of social capital and the importance of relationships within involvement. People with lived experience could access new involvement opportunities through the relationships they developed with researchers. A key aspect of these relationships is their reciprocal nature where advisors are not only giving something to research but also getting something in return [93]. Yet, the allocation of junior staff and students to deliver PPI activities means they have less experience and are on shorter contracts, impacting the ability to develop long‐term relationships and having less power to embed PPI and respond to feedback [94]. This results in contradictory practices where people are ‘invited in’ to PPI based on their skillset, but then these skills and knowledge are not used to their full capabilities.

Building on the exploration of symbolic capital in PPI in health research [95], thinking with social capital demonstrates the different forms of ‘capital’ that advisors bring to involvement. For example, lived experience can be used as a resource but struggles to be considered equal to professional experience. Advisors’ previous education, work and prior involvement experience also gave them more capital to be invited to take part in other opportunities. However, there is also the risk that advisors and their experiences are treated as commodities within research and involvement [96].

Additionally, the pressures of making PPI a funding requirement for researchers have proven to be unproductive due to the additional burden created on workload, time and resources [3, 97]. Yet, funding presents as a barrier to adequately budget for involvement activities and pay advisors for their time. It is also essential to budget for staff time, which can often be overlooked [98]. Challenges to paying people fairly and appropriately have only recently been explored including a lack of guidance and infrastructure to acknowledge contributions [61, 99, 100].

Furthermore, there is a lack of consideration placed on advisors' emotional and embodied experiences of PPI in relation to their health conditions. For example, having to retell painful or upsetting experiences, or the physical challenges of attending meetings. The emotional experience of PPI involves feelings of vulnerability where people with lived experience are exposed to the realities of their health through unwanted information or ‘information harm’ [101]. This builds on the limited research that involvement can have negative impacts, creating a ‘burden of involvement’ for advisors and researchers [2, 94, 102]. If this emotional labour is not acknowledged or supported appropriately by the research team, it may devalue advisors' efforts.

Finally, this review helped to understand researchers' published accounts of PPI as an activity and what they considered valuable to investigate and report. These accounts showed the tendency for PPI results to be reported positively in publications [103] and how challenges are easily minimised, under‐reported or not reported at all [104, 105]. Despite journals requiring authors to report on their PPI activities, recent studies have found reporting to be low and inconsistent in quality [106, 107, 108]. A scoping review of PPI in dementia research also found the researcher's perspective to be dominant, rather than the voice of PPI advisors despite being named as co‐authors [109]. Recent guidance considers how patient partners should be included in acknowledgements or as co‐authors [110].

## 5 Limitations

This review had several limitations. First, due to the chosen search terms and inconsistent variations in involvement terminology, some articles may have been missed. Although this review's broad inclusion criteria sought to capture a wide range of experiences and evidence, this resulted in a limited number of direct perspectives from patients and carers. However, this could be reflective of how patients' perspectives are included in published literature. Second, articles were limited to those published in English. The 40 articles where full text was not available and the exclusion of some commentaries in the initial abstract and title screening phase could have impacted the review's findings. We therefore included commentaries when we updated the review. Finally, publication, reporting biases and lack of detailed evidence may have also impacted the conclusions of this review. To address this, we published our scoping review protocol to increase transparency and used two reviewers to screen articles to reduce bias.

## 6 Conclusion

This review highlights important opportunities and challenges for improving how PPI is valued and practised beyond a process or method. It shows how valuing both advisors' and researchers' contributions can be an impact of involvement. The values of involvement, including relationship building, valuing experiential knowledge and on‐going communication are key to engaging meaningfully with advisors and researchers. Researchers need to recognise and support the different forms of capital that advisors bring in addition to their experiences. Emotional and physical challenges of involvement must be acknowledged and addressed through individualised support and flexibility. Finally, improving transparency and consistency of PPI reporting is crucial to ensure successes and challenges are from the perspective of advisors, and they are recognised and credited appropriately. Future research should focus on addressing the structural and relational challenges of embedding lived experience in research and how advisors' contributions can be valued equally to professional expertise to effectively improve how research is conducted.

## Author Contributions

Conceptualisation: Heather Mah, Alison Thomson, Ruth Dobson. Data curation: Heather Mah. Formal analysis: Heather Mah. Funding acquisition: Alison Thomson, Ruth Dobson. Investigation: Heather Mah, Alison Thomson, Ruth Dobson. Methodology: Heather Mah, Alison Thomson, Ruth Dobson. Supervision: Alison Thomson, Ruth Dobson. Validation: Heather Mah, Alison Thomson, Ruth Dobson. Writing – original draft: Heather Mah. Writing – review and editing: Heather Mah, Alison Thomson, Ruth Dobson.

## Ethics Statement

The authors have nothing to report.

## Conflicts of Interest

Heather Mah's PhD studentship is funded by the Horne Family Charitable Foundation.

Ruth Dobson has received honoraria for speaking and/or travelling from Biogen, Esai, Merck, Roche, Teva, Janssen and Sanofi. She served on the advisory board of Roche, Biogen, Janssen and Merck. She has received grant support from Biogen, Merck, Celgene, Barts Charity, the UK MS Society, NMSS, MRC and the Horne Family Charitable Foundation.

Alison Thomson has received grant support from the UK MS Society, Roche, Merck, Barts Charity, NIHR, ADR UK and honoraria from Novartis, Roche, Merck and NeuraxPharm.

## Supporting information

Supplementary Information

## Data Availability

The authors confirm that the data supporting the findings of this study are available within the article (and/or) its Supporting Information.
