# Supplementary material for: The Importance of Lived Experience: A Scoping Review on the Value of Patient and Public Involvement in Health Research
Source: Health Expect. 2025 Mar 26;28(2):e70205. doi: 10.1111/hex.70205 (PMC11938292; doi:10.1111/hex.70205)
Supplement: Supplementary file 1 — Supplementary Information [file HEX-28-e70205-s001.docx]

**Additional File 1: Preferred Reporting Items for Systematic reviews and Meta-Analyses extension for Scoping Reviews (PRISMA-ScR) Checklist**

| **SECTION** | **ITEM** | **PRISMA-ScR CHECKLIST ITEM** | **REPORTED ON PAGE #** |
| --- | --- | --- | --- |
| **TITLE** | | | |
| Title | 1 | Identify the report as a scoping review. | 1 |
| **ABSTRACT** | | | |
| Structured summary | 2 | Provide a structured summary that includes (as applicable): background, objectives, eligibility criteria, sources of evidence, charting methods, results, and conclusions that relate to the review questions and objectives. | 2 |
| **INTRODUCTION** | | | |
| Rationale | 3 | Describe the rationale for the review in the context of what is already known. Explain why the review questions/objectives lend themselves to a scoping review approach. | 5, 6 |
| Objectives | 4 | Provide an explicit statement of the questions and objectives being addressed with reference to their key elements (e.g., population or participants, concepts, and context) or other relevant key elements used to conceptualize the review questions and/or objectives. | 6 |
| **METHODS** | | | |
| Protocol and registration | 5 | Indicate whether a review protocol exists; state if and where it can be accessed (e.g., a Web address); and if available, provide registration information, including the registration number. | 6 |
| Eligibility criteria | 6 | Specify characteristics of the sources of evidence used as eligibility criteria (e.g., years considered, language, and publication status), and provide a rationale. | 6 |
| Information sources* | 7 | Describe all information sources in the search (e.g., databases with dates of coverage and contact with authors to identify additional sources), as well as the date the most recent search was executed. | 6 |
| Search | 8 | Present the full electronic search strategy for at least 1 database, including any limits used, such that it could be repeated. | 7 |
| Selection of sources of evidence | 9 | State the process for selecting sources of evidence (i.e., screening and eligibility) included in the scoping review. | 7 |
| Data charting process | 10 | Describe the methods of charting data from the included sources of evidence (e.g., calibrated forms or forms that have been tested by the team before their use, and whether data charting was done independently or in duplicate) and any processes for obtaining and confirming data from investigators. | N/A |
| Data items | 11 | List and define all variables for which data were sought and any assumptions and simplifications made. | N/A |
| Critical appraisal of individual sources of evidence | 12 | If done, provide a rationale for conducting a critical appraisal of included sources of evidence; describe the methods used and how this information was used in any data synthesis (if appropriate). | N/A |
| Synthesis of results | 13 | Describe the methods of handling and summarizing the data that were charted. | 7,8 |
| **RESULTS** | | | |
| Selection of sources of evidence | 14 | Give numbers of sources of evidence screened, assessed for eligibility, and included in the review, with reasons for exclusions at each stage, ideally using a flow diagram. | 8 |
| Characteristics of sources of evidence | 15 | For each source of evidence, present characteristics for which data were charted and provide the citations. | Additional file 2 |
| Critical appraisal within sources of evidence | 16 | If done, present data on critical appraisal of included sources of evidence (see item 12). | N/A |
| Results of individual sources of evidence | 17 | For each included source of evidence, present the relevant data that were charted that relate to the review questions and objectives. | Additional file 2 |
| Synthesis of results | 18 | Summarize and/or present the charting results as they relate to the review questions and objectives. | 8-13 |
| **DISCUSSION** | | | |
| Summary of evidence | 19 | Summarize the main results (including an overview of concepts, themes, and types of evidence available), link to the review questions and objectives, and consider the relevance to key groups. | 13,14 |
| Limitations | 20 | Discuss the limitations of the scoping review process. | 14 |
| Conclusions | 21 | Provide a general interpretation of the results with respect to the review questions and objectives, as well as potential implications and/or next steps. | 14,15 |
| **FUNDING** | | | |
| Funding | 22 | Describe sources of funding for the included sources of evidence, as well as sources of funding for the scoping review. Describe the role of the funders of the scoping review. | 1 |

**Additional File 2: Grey literature online searches**

| Organisation | Website | Comments | Definitions | Programmes | Resources |
| --- | --- | --- | --- | --- | --- |
| UK |  |  |  |  |  |
| Medical Research Council (UK Research and Innovation) | <https://www.ukri.org/councils/mrc/facilities-and-resources/find-an-mrc-facility-or-resource/mrc-regulatory-support-centre/understanding-health-research/patient-and-public-involvement/> | Refers to co-production, acknowledges accountability and power, discussing motivations and responsibilities, ongoing monitoring  Unclear why later calls PPI public involvement Signed shared commitment to improve PPI in HSC research Examples of good practice  Updated 2022 | People with lived experience (such as your participants or patients themselves, a group of people like them, or their carers) |  | - Signposts to NHS HRA  - Guidance on co-production  - References UK standards for public involvement |
| NIHR (National Institute of Health and Care Research) | <https://www.nihr.ac.uk/patients-carers-and-the-public/> | - Several different webpages available for members of the public but also resources and guidance for researchers | NIHR defines public involvement in research as research being carried out ‘with’ or ‘by’ members of the public rather than ‘to’, ‘about’ or ‘for’ them.  When we use the term ‘public’, we are including:  patients and potential patients  people who use health and social care services  carers  people from organisations that represent people who use services. | - Opportunities to join a research committee, be a study participant, become a reviewer or research champion | - Signposts to UK Standards of Public Involvement, research guidance, payment, giving feedback, co-production, evaluating and reporting PPI  - Public information pack available |
| USA |  |  |  |  |  |
| NIH (National Institute of Health) | <https://www.nih.gov/about-nih/what-we-do/get-involved-nih/public-involvement-nih> | Very limited information, web page difficult to find, last updated Feb 2020 - aimed for clinical research, unclear how to get involved, seems more about participating in trials. No guidance | Uses the term 'Public representatives' no definition provided | Volunteer opportunities: clinical trials, clinical centre, national history of medicine |  |
| PCORI (Patient-Centred Outcomes Research Institute) | <https://www.pcori.org/engagement/value-engagement> | Equitable partners vs research subjects | What is Engagement in Research?  The meaningful involvement of patients, caregivers, clinicians, and other healthcare stakeholders throughout the entire research process—from planning the study, to conducting the study, and disseminating study results. |  | Compensation Framework - 2015, no rates provided <https://www.pcori.org/sites/default/files/PCORI-Compensation-Framework-for-Engaged-Research-Partners.pdf> |
| National MS Society | <https://mssociety.ca/get-involved> | Research priorities - co-produced? Strategic plan - mentions 'collective action' but unclear what this actually means/ looks like in practice |  | MS Research Portal - participant opportunities |  |
| Australia |  |  |  |  |  |
| NHMRC (National Health and Medical Research Council) | <https://www.nhmrc.gov.au/about-us/consumer-and-community-engagement> | Public consultation - ad hoc  Representation on committees, unclear what membership involves or how to apply 2021-2024 term  Research committee - unclear who has lived experience, maybe one consumer representative with professional background? <https://www.nhmrc.gov.au/about-us/leadership-and-governance/committees/research-committee/research-committee-2021-2024> | Consumer and community engagement | Consumer and community advisory group made up of 12 for 3-year term  Grant peer review | Resources available to download - publication/version date not stated |
| CCIP (Consumer and Community Involvement Program) | <https://cciprogram.org/> | Good programmes and training available | Community involvement | CCIP program  Apply for involvement opportunities  Free training workshops: Intro to CCI, grant review panels, writing in plain language  Register for newsletter |  |
| Canada |  |  |  |  |  |
| CIHR (Canadian Institutes of Health Research) | <https://cihr-irsc.gc.ca/e/41592.html> | Some web pages not updated since 2012  selection criteria - doesn't include lived experience, requires researchnet account to apply | CIHR has adopted the term citizen engagement because the essence of "engagement" is far more active than traditionally passive public consultation in its recognition of the capacity of citizens to discuss and generate options independently. The term "citizen" includes interested representatives from the general public, consumers of health services, patients, caregivers, advocates, and representatives from affected community and voluntary health organizations | Institute Advisory Board Member | terms of reference, selection criteria, 8-14 members, terms of three years, max 6 years, voluntary, expenses only  Citizen Engagement Framework 2012 |
| SPOR (Strategy for Patient-Oriented Research) | <https://cihr-irsc.gc.ca/e/41204.html> |  | For SPOR, the term patient is overarching and is inclusive of individuals with personal experience of a health issue and informal caregivers, including family and friends.  patient representatives, patient engagement experts | Research networks support units | SPOR patient engagement framework - patient input, date not stated  Compensation <https://cihr-irsc.gc.ca/e/51466.html>  Newsletter |

**Additional File 3: Table of Included Grey Literature Resources**

| First Author | Title | Year | Country of Origin | Type of Resource | Health Condition |
| --- | --- | --- | --- | --- | --- |
| Arthritis Research UK | Patient and Public Involvement A Researcher’s Guide | No Date | United Kingdom | Guidance | Arthritis |
| Canadian Institutes of Health Research | Strategy for Patient-Oriented Research Patient Engagement Framework | 2014 | Canada | Co-produced Framework | Not health condition specific |
| European Patients’ Forum | The Value+ Toolkit. | No Date | Europe | Co-developed Toolkit | Not health condition specific |
| Hanley, B. | Patient and public involvement in  laboratory based research:  Reflections on six studies | 2020 | United Kingdom | Report | Alzheimer's, Parkinson’s |
| Health Service Executive Research and Development | Knowledge Translation, Dissemination, and Impact: A Practical Guide for Researchers. | 2021 | United Kingdom | Guidance | Not health condition specific |
| Newman | PIRIT Public Involvement in Research Impact Toolkit | 2023 | Wales | Co-developed Toolkit | Cancer |
| NIHR Imperial BRC Patient Experience Research Centre | A Rough Guide to Public Involvement | 2021 | United Kingdom | Guidance | Not health condition specific |
| People in Health West of England | PPI Impact Log | 2019 | United Kingdom | Co-developed Impact Log | Not health condition specific |
| Turk, A. | A Researcher’s Guide to Patient and Public Involvement | 2017 | United Kingdom | Guidance | Not health condition specific |

**Additional File 4: Summary of key findings**

| **Theme 1: Value from contributing to research** |
| --- |
| - **Gaining new skills, knowledge and accessing opportunities** (Bayliss, 2017; Evans et al., 2014; Giebel et al., 2023; Mitchell et al, 2021; Saini, 2021; Simpson et al., 2014; Slade et al., 2016) - **Increasing knowledge about health condition, treatment and causes** (Dwyer et al., 2021; Gutman et al., 2020; Hemphill et al., 2019; Hough et al., 2024; Hoven et al., 2020; Musson et al., 2019; Schilling et al., 2019b) - **Altruistically motivated to help others and improve future health care and research** (Hemphill et al., 2019; Hough et al., 2024; Jorgenson et al., 2018; Milley et al., 2021) - **Helping to change career, return to work or education** (Ashcroft et al., 2016; Foster et al., 2021; Hemphill et al., 2019; Simpson et al., 2014) - **Providing a sense of belonging and purpose** (Booker-Vaughns et al., 2024; Evans, 2014; Gordon et al., 2018; Hemphill et al., 2019; Hough et al., 2024; Hovered et al., 2024; Locock et al., 2019; Merker et al., 2022; Mockford et al., 2016; Patterson et al., 2014) - **Paying for their time** (Belisle-Pino et al., 2021; de Wit et al., 2023; Dhamanaskar et al., 2024; Simpson et al., 2014) - **Co-authoring journal articles** (de Wit et al., 2023; Di Lorito et al., 2020; Elliott et al., 2023; Oliver et al., 2020) - **Actively involved in discussions, provided regular feedback and communication** (Evans et al., 2014; Fleming et al., 2021; Meudell et al., 2017; Mitchell et al., 2020) |
| **Theme 2: Importance of relationships** |
| - **Developing nurturing, trusting, respectful relationships** (Birch et al., 2020; Booker-Vaughns et al., 2024; Carroll et al., 2024b; Di Lorito et al., 2020; Evans, 2014; Foster et al., 2021; Hoverd et al., 2024; Kwok et al., 2022; Luna Puerta et al., 2020; Mann et al., 2018; Mitchell et al., 2020; Pritchard et al., 2024; Slade et al., 2016; Schandl et al., 2022; Smith et al., 2024; Tripp et al., 2023) - **Patients are treated as equal members of the team** (Bradshaw et al., 2021; Evans et al., 2022; Green & Johns, 2019; Gamble et al., 2015; Garfield et al., 2015; Hoven et al., 2020; Mitchell et al., 2020; Oliver et al., 2020; Richmond et al., 2023) - **Contributions are recognised, taken seriously and appreciated** (Elliott et al., 2023; Milley et al. 2021; Saini et al., 2021; Shilling et al., 2019b; Warner et al., 2021) - **Developing relationships take time** (Carroll et al., 2024a; Di Lorito et al., 2020; Hoven et al., 2020) |
| **Theme 3: Attitudes and Support for PPI** |
| - **Lack of effective institutional processes or adequate budgeting to pay people for their time** (Beland et al., 2022; Brighton et al., 2018; Di Lorito et al., 2020; Jorgenson et al., 2018Tremblay et al., 2020; Vat et al., 2020) - **Experiential knowledge was not always recognised, valued or given “equal status”** (Ashcroft et al., 2016; Di Lorito, 2020; Fleming et al., 2021; Green & Johns, 2019; Patterson et al., 2014) - **Negative attitudes towards PPI** (Ashcroft et al., 2016; Green & Johns, 2019; Harmsen et al., 2022; Patterson et al., 2014) |
| **Theme 4: Emotional Labour of Involvement** |
| - **Negative physical and emotional demands** (Evans, 2014; Mitchell et al., 2020; Musson et al., 2019; Richmond et al., 2023; Schilling et al., 2019a; Vat et al., 2020) - **Fears about their health and future** (Carroll et al., 2024b; Musson et al., 2019) - **Sharing experiences enables personal growth** (Gutman et al., 2022; Horton et al., 2021; Richards et al., 2023) |

**Additional File 5: Mapping of Principles with the UK PPI Standards**

| **UK Standards for Public Involvement (2019)** | **Working together** | **Inclusive Opportunities** | **Impact** | **Governance** | **Communica-tion** | **Support and Training** |
| --- | --- | --- | --- | --- | --- | --- |
| Arthritis Research UK (n.d.) | Give it Time, Be Considerate, Manage Expectations | Diversity | Evaluate | Consider Managerial Roles | Feedback | Budget, Recognition |
| Hanley, 2020 | Building a Relationship, Small Numbers, Face-to-face Meetings, Clarity of Purpose | Involving the ‘right’ people |  |  | Preparation, Keeping Contributors Informed | Support, Commitment and Interest, |
| Turk et al., 2017 | Creating a good atmosphere, long-lasting relationships, Clarity |  |  | Record-keeping, good chairing | Feedback | Training, payment of expenses, catering |
| CIHR, 2014 | Shared sense of purpose, experiential knowledge valued as evidence | Inclusive mechanisms and processes |  | Patient-informed and directed research | Multi-way communication and collaboration | Support, multi-way capacity building |
| PCORI, 2024 | Building capacity to work as a team | Diversity and representation | Ongoing review and assessment of engagement | Meaningful inclusion of partners in decision making | Early and ongoing engagement | Dedicated funds for engagement and compensation |
| HSE, 2021 | Respect, Manage expectations | Accessible | Needs assessment | Communication, preparation for activities | Regular feedback, payment | Support and training |
| NIHR BRC, 2021) | Managing expectations | Supporting diversity and inclusion |  |  |  | Providing training, rewarding for time |

**Additional File 6: Table of included studies**

| First Author | Title | Year | Country | Aim of study | Study design |
| --- | --- | --- | --- | --- | --- |
| Ashcroft | Impact on the individual: what do patients and carers gain, lose and expect from being involved in research? | 2016 | United Kingdom | This study explored how patients and carers in eight diagnostic research specialties have been involved in research, their motivations and the impact involvement had on them. | This study was a mixed method, cross-sectional self-report survey, utilising a semi-structured questionnaire containing closed questions and text boxes for further explanation. |
| Barn | Better Together  Launching and Nurturing a Community Stakeholder Committee to Enhance Care and Research for Asthma and COPD | 2022 | Canada | To describe experience with creating and  developing an ongoing Community Stakeholder Committee to guide lung health research for disease prevention and health care improvement. | A baseline evaluation survey after 1 year (October 2020), using a modified version of the Patient Engagement in Research Scale (PEIRS-22). |
| Barrett | Evaluating parent and public involvement activities within a paediatric palliative care research centre: Route map to impactful and meaningful engagement | 2024 | United Kingdom | To evaluate a paediatric palliative care research centre’s PPI activity to determine what went well, or less well; and how future activities can be improved. | Two stage evaluation: first a review of PPI study logs; second a qualitative exploration using a survey, structured interviews and a focus group. Data were analysed thematically. |
| Bayliss | Patient involvement in a qualitative meta-synthesis: lessons learnt | 2016 | United Kingdom | To inform the evidence base on effective ways of involving patients in a qualitative meta-synthesis. | An open-ended questionnaire (available from the authors) was developed to collect data about the PRP’s experience of collaborating with researchers to complete a qualitative meta-synthesis. |
| Bayliss | Working with public contributors to improve the patient experience at the Manchester Clinical Research Facility: an evaluation of the Experience Based Design approach | 2017 | United Kingdom | To evaluate the Experience Based Design (EBD) process from a public involvement perspective, exploring the barriers and facilitators to building successful working relationship. | An open-ended evaluation questionnaire was developed to gain feedback from staff and public contributors who co-produced an EBD project that aimed to improve the patient experience at Manchester CRF. |
| Beland | Patient and researcher experiences of patient engagement in primary care health care research: A participatory qualitative study | 2022 | Canada | To describe and compare patient partners' and academic researchers' experiences in patient engagement research. | Individual semi-structured interviews with patient partners (n = 7) and academic researchers (n = 15) were conducted. |
| Belisle-Pipon | Equality and Equity in Compensating Patient Engagement in Research: A Plea for Exceptionalism | 2021 | Canada | To argue that there are good reasons for exceptionalism and that clearer guidance on citizen/patient engagement in research should be embedded into research ethics doctrine. | Commentary on experiences of obtaining an ethics certificate from an institutional review board (IRB) to conduct a study that places citizen/patient perspectives on equal footing with those of academic/policy experts. |
| Birch | Development and formative evaluation of patient research partner involvement in a multi-disciplinary European translational research project | 2020 | United Kingdom | To describe the development of PPI in the EuroTEAM project, and the results of two mixed-methods surveys. | Two mixed-methods surveys quantitative and qualitative (for PRPs and researchers) and a teleconference were undertaken to assess the impact of PPI on individual work packages and on EuroTEAM overall. A teleconference was held with PRPs and researchers to review and interpret the survey findings and their implications for future projects. |
| Booker-Vaughns | What's in This For You? What's in This For Me?: A Win-Win Perspective of Involving Study Advisory Committee Members in Palliative Care Research | 2024 | USA | We use reflective dialogue to share SAC members’ lived experiences and the impact the EMPallA study has had on members both personally and professionally. | Narrative on experiences of Emergency Medicine Palliative Care Access (EMPallA) study. |
| Bradshaw | Value, transparency, and inclusion: A values based study of patient involvement in musculoskeletal research | 2021 | United Kingdom | To use a values-based approach to reporting PPI at a Research Unit focused on musculoskeletal conditions within a UK medical school. | Participants completed a structured questionnaire based on the value system framework; PPI members also provided further information through telephone interviews. |
| Brett | Impact of patient and public (PPI) involvement in the Life After Prostate Cancer Diagnosis (LAPCD) study: a mixed-methods study | 2022 | United Kingdom | To explore the impact of PPI in a large UK study, the Life After Prostate Cancer Diagnosis (LAPCD) study, and to explore the facilitators and challenges experienced. | Mixed-methods study using an online survey and semi structured interviews. |
| Brighton | Taking patient and public involvement online: qualitative evaluation of an online forum for palliative care and rehabilitation research | 2018 | United Kingdom | In this study, we explored how well the online forum worked, if it is a suitable method for PPI, and how PPI members and researchers reacted to using it. | We conducted separate semi-structured face-to-face qualitative focus groups with PPI members and researchers who had used the online forum. Three focus groups held - one with researchers, two with PPI members. Reported in line with COREQ. |
| Brown | Openness, inclusion and transparency in the practice of public involvement in research: A reflective exercise to develop best practice recommendations | 2018 | United Kingdom | To critically reflect on the PI practices that underpinned our research project on intimacy and sexuality in care homes, to develop a series of recommendations for improving future PI activities. | Reflexive exercise on recent PI project. Each CR interviewed individually by academic team members. Academic team member completed written responses to 5 open ended questions. Views discussed at workshop. |
| Buck | From plans to actions in patient and public involvement: Qualitative study of documented plans and the accounts of researchers and patients sampled from a cohort of clinical trials | 2014 | United Kingdom | Inform the evidence base by describing how plans for PPI were implemented within clinical trials and identifying the challenges and lessons learnt by research teams. | "We compared PPI plans extracted from clinical trial grant applications (funded by the National Institute for Health Research Health Technology Assessment Programme between 2006 and 2010) with researchers and PPI contributors‚ interview accounts of PPI implementation. This qualitative study formed part of the 'Evidence base for Patient and public Involvement in Clinical trials' (EPIC) project. |
| Carlsson | Being involved in research as a collaborator with experience of a prenatal diagnosis of congenital heart defect in the fetus: a qualitative study | 2020 | Sweden | To explore how persons with lived experience of a prenatal diagnosis perceived collaborating in a research project utilizing patient and public involvement to identify relevant research questions and develop suitable interventions. | This was a qualitative interview study. Two groups were formed that had consecutive meetings during the yearlong project: one consisting of parents of living children prenatally diagnosed with a congenital heart disease (n=5) and another group consisting of persons who terminated the pregnancy following the diagnosis (n= 5). Follow-up telephone interviews about experiences of the project. |
| Carroll | Perspectives of researchers and clinicians on patient and public involvement (PPI) in preclinical spinal cord research: An interview study | 2024a | Ireland | To explore the perspectives of two key stakeholder groups, preclinical researchers and clinicians on PPI in preclinical research, using spinal cord research as a case study. | Semi-structured interviews were conducted online with 11 clinicians and 11 preclinical researchers |
| Carroll | The role of patient and public involvement (PPI) in pre-clinical spinal cord research: An interview study | 2024b | Ireland | To explore the perspectives of seriously injured rugby players’ who live with a spinal cord injury on PPI in pre-clinical research. | Semi-structured interviews were conducted via telephone with 11 seriously injured rugby  players living with spinal cord injury on the island of Ireland. |
| Chudyk | Future directions for patient engagement in research: a participatory workshop with Canadian patient partners and academic researchers | 2024 | Canada | To identify future directions for Canadian patient engagement in research and discusses its findings in the context of the international literature. | The study met its aim through a multi-meeting pan-Canadian virtual workshop. The workshop was composed of four, 1.5–3-h virtual meetings wherein participants discussed the current and preferred future states of Canadian patient engagement in research. |
| Chung | Crohn's and Colitis Foundation of America Partners Patient-Powered Research Network: Patient Perspectives on Facilitators and Barriers to Building an Impactful Patient-Powered Research Network | 2018 | United States | To better understand patients' preferences for what are the essential features that will facilitate and sustain engagement. | We conducted a two-phase qualitative study. Seven focus groups involving 62 participants with IBD were conducted (phase1). Focus group results informed the phase 2 cognitive interviews, which included 13 phone interviews. Topics included experiences with IBD and research, PPRN engagement, patient-generated health data, and resources/tools to facilitate self-management. |
| Coupe | Patient and public involvement in doctoral research: Impact, resources and recommendations | 2019 | United Kingdom | To explore the impact of PPI on two health-related doctoral research studies and identify how PPI could be used meaningfully at this level. | The PPI processes included (a) involvement of two 'Research Buddies' who informed the research design and ensured implementation of a booklet intervention was feasible for family carers, (b) data analysis workshops with 'Research Buddies' to identify emerging themes from practitioner interviews, (c) public and stakeholder involvement who informed data collection tool design, and the design of an intervention to help people with obesity who attend weight loss groups. |
| Dawes | Capturing learning from public involvement with people experiencing homelessness to help shape new physiotherapy research: Utilizing a reflective model with an under-served, vulnerable population | 2022 | United Kingdom | To impart learning to help inform researchers undertaking PPI with other underserved and vulnerable groups. | Six people with experience of homelessness attended a PPI consultation aided by Pathway, a UK homeless peer advocacy charity, which coordinates an ‘Experts by Experience’ group. |
| Dawson | Patient and public involvement in doctoral research: reflections and experiences of the PPI contributors and researcher | 2020 | United Kingdom | To address a gap in the literature by providing an exemplar that focuses on the journey of PPI within this doctoral research. | A reflective approach was taken using data from PPI contributor and researcher notes, e-mail correspondence, meeting notes. |
| de Groot | Meaningful Engagement of Persons Affected by Leprosy in Research: An Exploration of Its Interpretation, Barriers, and Opportunities | 2023 | Netherlands | To explore the interpretation, barriers and opportunities of meaningful engagement of persons affected by leprosy in research. | (i) an exploratory phase consisting of key informant interviews with experts in public and patient involvement (n = 2) and experts-by-experience (i.e., persons affected by leprosy; n = 4), and (ii) an in-depth phase among leprosy researchers consisting of an online survey (n = 21) and key informant interviews (n = 7). |
| De Simoni | Patient and public involvement in research: the need for budgeting PPI staff costs in funding applications | 2023 | United Kingdom | To explore how to how to cost PPI staff time in funding applications | Reflection on how the Asthma UK Centre for Applied Research (AUKCAR) has organised staff to support for its patient involvement. |
| de Wit | Involving patient research partners has a significant impact on outcomes research: a responsive evaluation of the international OMERACT conferences | 2013 | Netherlands | To assess the inclusion of patients as international research partners in Outcome Measures in Rheumatology (OMERACT) conferences and how this has influenced the scope and conduct of outcomes research in rheumatology. | A thematic content analysis of OMERACT internal documents, publications and conference proceedings, followed by a responsive evaluation including 32 qualitative semi structured interviews. |
| de Wit | Successful stepwise development of patient research partnership: 14 years experience of actions and consequences in Outcome Measures in Rheumatology (OMERACT) | 2017 | Netherlands | Based on a review of cumulative data from qualitative studies and internal surveys among OMERACT participants, we explored the potential benefits and challenges of involving patient research partners in conferences and working group activities. | Based on a review of cumulative data from qualitative studies and internal surveys among OMERACT participants. |
| de Wit | EULAR recommendations for the involvement of patient research partners in rheumatology research: 2023 update | 2023 | Netherlands | Since the publication of the 2011 European Alliance of Associations for Rheumatology (EULAR) recommendations for patient research partner (PRP) involvement in rheumatology research, the role of PRPs has evolved considerably. Therefore, an update of the 2011 recommendations was deemed necessary. | The process included an online task force meeting, a systematic literature review and an in-person second task force meeting to formulate overarching principles (OAPs) and recommendations. |
| Dhamanaskar | Patient partner perspectives on compensation: Insights from the Canadian Patient Partner Survey | 2024 | Canada | To describe the types and frequency of compensation patient partners have been offered and their attitudes towards compensation. | This study uses data from the Canadian Patient Partner Study (CPPS) survey. The survey gathered the experiences and perspectives of those who self-identified as patient partners working across the Canadian health system. |
| Di Lorito | Adding to the knowledge on patient and public involvement: Reflections from an experience of co-research with carers of people with dementia | 2020 | United Kingdom | To propose a model for good practice in co-researching with carers of people with dementia, by reporting and synthesizing the personal reflections of the academic and lay researchers around the methodological issues, benefits, and challenges of co-research. | The participants and their carers were interviewed (as dyads) through qualitative semi-structured interviews in their private home. |
| Dovey-Pearce | The burden of proof: The process of involving young people in research | 2019 | United Kingdom | This study describes the experiences of adult researchers and young people involved in a large-scale, UK health research programme, exploring the process of working together and the outcomes of that work. | Qualitative interviews with the senior academic researchers and involvement facilitators from the Transition programme were carried out (n = 10). One person (the involvement lead) carried out the interviews. Four iterative focus groups with UP Group members took place. |
| Dwyer | A qualitative investigation of reasoning behind decisions to decline participation in a research intervention: A study-within-a-trial | 2021 | Ireland | The aims of this qualitative study-within-a-trial (SWAT) is to explore individuals' decisions to decline participation in a research programme trailing a therapy related to their chronic illness (see Study Context & Design) and to extend research on recruitment, which may, in turn, enhance the development and administration of future RCTs. | A series of semi-structured, one-on-one tele-phone interviews were conducted to explore decliners' reasons not to participate in the host trial, as well as their perception of the research. |
| Elliott | The dynamic nature of patient engagement within a canadian patient‐oriented kidney health research network: Perspectives of researchers and patient partners | 2023 | Canada | To explore how research teams integrated patient partners into network research activities to inform our patient engagement approach | This qualitative descriptive study purposively sampled researchers and patient partners across 18 network research teams. We conducted 4 focus groups (2 patients and 2 researchers; n = 26) and 28 individual telephone interviews (n = 12 patient partners; n = 16 researchers). |
| Evans | Patient and public involvement in research in the English NHS: a documentary analysis of the complex interplay of evidence and policy. | 2014 | United Kingdom | The aim was to identify the contextual factors and mechanisms that are regularly associated with effective public involvement in research. | The objectives included identifying a sample of eight research projects and their desired outcomes of public involvement, tracking the impact of public involvement in these case studies, and comparing the associated contextual factors and mechanisms. |
| Evans | Between funder requirements and 'jobbing scientists': the evolution of patient and public involvement in a mental health biomedical research centre - a qualitative study | 2020 | United Kingdom | The aim of the study was to explore the development of PPI within a London based mental health biomedical research centre (BRC) over a period of 10 years from its inception. | This retrospective qualitative study compared data from 52 organisational documents and 16 semi-structured interviews with staff and service users associated with PPI within the Maudsley BRC. |
| Evans | Implementing public involvement throughout the research process—experience and learning from the gps in eds study | 2022 | United Kingdom | To describe how we implemented public involvement, reflect on process and effects in a large-scale multi-site research study and present learning for future involvement practice | We recorded public involvement roles and activities throughout the study and compared these to our original public involvement plan included in our project proposal. We held a group interview with study co-applicants to explore their experiences. |
| Fleming | Patient engagement in fertility research: bench research, ethics, and social justice | 2021 | United States | We described PPI activities in the beginning stages of a research project and evaluated the PESMs engagement in a basic science study seeking to improve IVF out-comes. | Subsequent evaluation of engagement efforts occurred by reviewing the PES reports for the Embryo+ researchers, conducting two recorded web-based discussion meetings with the PES (summer 2020; meeting 1n= 7; meeting 2n= 6), and a brief survey (n= 13). |
| Foster | "PPI? That sounds like Payment Protection Insurance": Reflections and learning from a substance use and homelessness study Experts by Experience group | 2021 | United Kingdom | To describe the activities and lessons learned from the involvement of an Experts by Experience group in a National Institute for Health Research (NIHR) study | This article describes the benefits of having Experts by Experience members as part of the project from the inception through to the end. |
| Gamble | An evidence base to optimise methods for involving patient and public contributors in clinical trials: a mixed-methods study | 2015 | United Kingdom | To establish an unselected cohort of randomised trials to: 1. examine how PPI has been implemented and identify associated impact 2. systematically describe and critically evaluate the process, challenges and impact of PPI from the perspectives of the PPI contributors, chief investigator (CI) and clinical trials unit (CTU) staff. | Using mixed-methods research we investigated PPI from the perspectives of researchers and PPI contributors. Chief investigators (CIs), PPI contributors and UK Clinical Research Collaboration Registered Clinical Trials Units (RCTUs) were surveyed. Interviews were conducted with researchers and PPI contributors. |
| Garfield | Patient and public involvement in data collection for health services research: a descriptive study | 2015 | United Kingdom | We wanted to explore the benefits and challenges of having lay people conduct these observations, to tell us more about how lay people can be involved in conducting such research. | We conducted semi-structured interviews with the lay members who collected observational data in our wider study and the researchers who provided support and/or were involved in their recruitment and training. |
| Garfield | Lay involvement in the analysis of qualitative data in health services research: a descriptive study | 2016 | United Kingdom | To report on our experiences with lay partners helping to conduct data analysis. | We conducted semi-structured interviews with the lay partners and researchers involved in qualitative data analysis in a wider study of inpatient involvement in medication safety. |
| Gheduzzi | How to prevent and avoid barriers in co-production with family carers living in rural and remote area: an Italian case study | 2021 | Italy | This paper investigates how the barriers that may arise during the co-production of a new social service with family carers can be identified and interpreted. | To investigate this topic, we undertook a single case study - a longitudinal project intended to co-produce a new social care service with and for the family carers of elderly patients living in rural and remote areas. |
| Glebel | A Community of Practice to increase education and collaboration in dementia and ageing research and care: The Liverpool Dementia & Ageing Research Forum | 2023 | United Kingdom | To outline the aims, components and evaluation of the public-facing and -engaging Liverpool Dementia & Ageing Research Forum, to provide a blueprint for setting up similar communities of practice. | All Forum members and attendees were asked to complete a brief evaluation survey about their experiences from October to November 2022. Three regular Forum attendees provided a case study about their involvement and its impact. |
| Giel | Enriching a randomized controlled treatment trial for anorexia nervosa by lived experience—Chances and effects of a lived experience council in the SUSTAIN study | 2024 | Germany | The present work outlines the participatory collaboration with a lived experience council in the randomized controlled treatment trial SUSTAIN. | In order to analyze possible effects of the LEC work, an electronic census was conducted among all LEC members 2.5 years after the project started. For this purpose, a questionnaire was designed by a subgroup of LEC members and the study team. |
| Gordon | Service user reflections on the impact of involvement in research | 2018 | United Kingdom | This commentary, written by two members of a hospital-based patient panel and their coordinator for its work, describes how we co-produced instruments to evaluate the impact and effectiveness of our involvement. | This commentary, written by two members of a hospital-based patient panel and their coordinator for its work, describes how we co-produced instruments to evaluate the impact and effectiveness of our involvement. |
| Green | Exploring the Relationship (and Power Dynamic) Between Researchers and Public Partners Working Together in Applied Health Research Teams | 2019 | United Kingdom | Conducting secondary analysis of a commissioned data set generated from semi-structured interviews with researchers and public partners, identified by PI experts as being demonstrably inclusive in their research, to see how this plays out in practice. | Secondary analysis of a commissioned data set generated from semi-structured interviews. |
| Griffiths | Reflections on co-production: Developing a dementia research funding application with a diverse lived experience group | 2024 | United Kingdom | To provide an example of how we co-produced a dementia research funding application and to share lessons learned in the process, to inform other researchers and non-researchers who are thinking of getting involved in co-production. | We reflected on our experiences of diversity and inclusion within the group, based on a coproduced set of questions to guide reflection. |
| Groot | What Patients Prioritize for Research to Improve Their Lives and How Their Priorities Get Dismissed again | 2022 | Netherlands | To conduct participatory research on patient experiences to create a solid research agenda with patients and discuss it with relevant stakeholders. | We followed a participatory research design in 18 sub-studies, including interviews and group sessions (n = 404 patients), and dialogue sessions (n = 367 professionals and directors in healthcare and social work, municipality civil servants, and funding agencies) on patient experiences with psychiatric care, community care, daycare, public health, and social work. |
| Gutman | Principles and strategies for involving patients in research in chronic kidney disease: report from national workshops | 2020 | Australia | We aimed to understand and describe patient and health professional perspectives about patient involvement in research in CKD and to identify practical solutions to engage and involve patients across the spectrum of BEAT-CKD research initiatives. | We convened workshops in three Australian cities involving 105 patients/caregivers and 43 clinicians/researchers. In facilitated breakout groups, participants discussed principles and strategies for effective patient involvement in chronic kidney disease research. |
| Gutman | Patient and Caregiver Experiences and Attitudes about Their Involvement in Research in Chronic Kidney Disease | 2022 | Australia | To describe patient and caregiver experiences and perspectives of their previous involvement in research, to inform strategies to strengthen patient involvement in research. | Semistructured interviews were conducted with 23 adult patients with CKD and caregivers who had previously been involved in research, from Australia, the United States, the United Kingdom, and Denmark |
| Harmsen | Supporting health researchers to realize meaningful patient involvement in research: Exploring researchers' experiences and needs | 2022 | Netherlands | (1) to gain deeper insight into the experiences and needs of researchers regarding meaningful patient involvement and (2) to incorporate these insights into an online tool. | This was done in a transdisciplinary research process, including three focus group discussions and three test sessions. |
| Hemphill | What motivates patients and caregivers to engage in health research and how engagement affects their lives: Qualitative survey findings | 2019 | United States | To inform effective engagement practices and policies, we sought to understand what motivates patients and caregivers to engage as partners on PCORI-funded research projects and how such engagement changed their lives. | We conducted thematic analysis of open-ended survey responses from 255 patients, family caregivers and individuals from advocacy and community-based organizations who engaged as partners on 139 PCORI-funded research projects focusing on a range of health conditions |
| Hickey | What Does "Good" Community and Public Engagement Look Like? Developing Relationships With Community Members in Global Health Research | 2022 | United Kingdom | To build on existing guidance and is a first step in trying to identify the key components of what “good” CPE looks like, which can be used for all approaches to global health research and in a range of different settings and contexts. | This article draws on data collected as part of an evaluation of CPE by 53 NIHR-funded award holders to provide insights on CPE practice in global health research. |
| Hobbs | Realist evaluation of public engagement and involvement in data-intensive health research | 2020 | United Kingdom | To evaluate if and how membership of a data-intensive research public forum can act as a mechanism for enhancing members personal development. | Qualitative data were collected from 15 current and previous members, via semi-structured interviews, notes from meetings, and consultations with and feedback from members. |
| Horton | In it for the long haul: a reflective account of collaborative involvement in aphasia research and education | 2021 | United Kingdom | To reflect on and learn from the experience of collaboration, examining the ways in which the involvement of people with aphasia and family members was carried out over a fifteen-year period in the context of a UK university. | We have taken a reflective longitudinal case study approach, and used constructs from implementation theory as sensitizing concepts in a cross-sectional analysis of documents. |
| Hough | Patient and public involvement and engagement (PPIE): how valuable and how hard? An evaluation of ALL_EARS@UoS PPIE group, 18 months on | 2024 | United Kingdom | To evaluate (1) the engagement, outputs, and outcomes of the PPIE group by monitoring activities over time (2) the impact, effectiveness, and engagement of the PPIE group ALL_EARS@UoS using an anonymous questionnaire. | An anonymous, mixed-methods questionnaire was co-designed and shared with members of ALL_EARS@UoS using an online platform. |
| Hoven | What makes it work? Exploring experiences of patient research partners and researchers involved in a long-term co-creative research collaboration | 2020 | Sweden | The aim of this study was to explore the experiences of patient research partners (PRPs) and researchers engaged in a co-creative long-term collaboration in cancer research. | The participants (11 PRPs, 6 researchers) took part in semi-structured telephone interviews. |
| Hoverd | Co-producing an online patient public community research hub: a qualitative study exploring the perspectives of national institute for health research (NIHR) research champions in England | 2024 | United Kingdom | To develop a university website for patients and members of the public to learn about ways to get actively involved in research and be able to access the results of health and social care research. | Content of a prototype Patient Public Community Research Hub website was co-produced with the Research Champions, and then 15 NIHR Research Champions from across England were asked for their views about the website. |
| Howells | Demonstrating the learning and impact of embedding participant involvement in a pandemic research study: the experience of the SARS-CoV-2 immunity & reinfection evaluation (SIREN) study UK, 2020-2023 | 2023 | United Kingdom | This paper aims to outline our approach to participant involvement in a large, multicentre pandemic response cohort study, providing a narrative account co-produced by PIP members and researchers. | To evaluate how the PIP has influenced SIREN, feedback was collected from (a) researchers presenting at the PIP and (b) PIP members themselves. |
| Jacob | From testers to cocreators—The value of and approaches to successful patient engagement in the development of eHealth solutions: Qualitative expert interview study | 2022 | Switzerland | To better understand the challenges  and opportunities in the inclusion of patients in the development  of eHealth technologies and ideas on how to overcome the identified gaps. | Key informant interviews were conducted using in-depth semi structured interviews with 20 participants from 6  countries across Europe. |
| Jenkins | Involving lived experience in regional efforts to address gambling-related harms: going beyond 'window dressing' and 'tick box exercises' | 2024 | United Kingdom | To discuss the role of LE in a UK city-region government’s gambling harms reduction intervention. | Three focus groups and 33 semi-structured interviews were conducted to hear from people with and without LE who were involved in the gambling harms reduction intervention, or who had previous experience of LE-informed efforts for addressing gambling-related harms. |
| Jorgensen | User involvement in a Danish project on the empowerment of cancer patients - experiences and early recommendations for further practice | 2018 | Denmark | The aim of the workshop was to explore the experiences of both academic researchers and co-researchers and through this, to provide early recommendations for the further development of user-involvement in the Danish context. | The workshop held with the academic researchers and co-researchers consisted of two parallel focus groups and a joint group discussion, following an interactive and informal format to facilitate discussion and exchange of ideas. |
| Kaisler | Enabling transdisciplinary collaboration: Stakeholder views on working with 'children with mentally ill parents' research groups | 2021 | Austria | To identify how stakeholders perceive transdisciplinary collaborations with researchers. | We conducted an online survey and interviews with the members of the advisory board and competence group. |
| Kilgour | Participating together in CP-ACHIEVE: Experiences, opportunities and reflections from a collaborative research team of people with lived experience of cerebral palsy and health care professionals | 2024 | Australia | To describe the CP-ACHIEVE values, structure and strategy for this approach, and its implementation at each stage of the research process. | We then provide an example of the strategy in action, using a qualitative exploration of CP-ACHIEVE’s Participation Theme team’s experiences of collaboration and involvement as co-researchers. |
| Kwok | Stroke survivors partner in research: a case example of collaborative processes | 2022 | Canada | To provide a case example of working together with three individuals who bring their post-stroke lived experience. | Detail how we were able to purposefully engage and include patient partners with post-stroke activity and communication limitations in a research project. |
| Laidlaw | Values and value in patient and public involvement: moving beyond methods | 2022 | United Kingdom | We argue that the conversation needs to change to one of ‘value’: a culture of common values and principles across all types of research. | We reflect on how our team's experiences as patients, healthcare professionals and academics have changed over time and shaped what we value, our involvement in research and the way we involve people in research. |
| Lampa | Tracking involvement over time: A longitudinal study of experiences among refugee parents involved as public contributors in health research | 2022 | Sweden | To track the experiences and perceived impact of refugee parents during their involvement as public contributors in a three-year child mental health trial. | The study used a longitudinal qualitative design with focus group discussions. |
| Lindberg | Engaging Patient Advisory Committees to Inform a Genomic Cancer Risk Study: Lessons for Future Efforts | 2022 | USA | To learn how patient stakeholders viewed the process, to inform future patient engagement efforts. | Using surveys and exit interviews, we evaluated stakeholders’ experiences as PAC members. |
| Lindenmeyer | Assessment of the benefits of user involvement in health research from the Warwick Diabetes Care Research User Group: a qualitative case study | 2007 | United Kingdom | To assess the benefits of involving health-care users in diabetes research. | For this qualitative case study, semi-structured interviews were conducted with researchers who had worked extensively with the group. Discussions during meetings, consulted external researchers, meeting minutes. |
| Locock | Involving service users in the qualitative analysis of patient narratives to support healthcare quality improvement | 2019 | United Kingdom | The co-authors were awarded a grant for secondary data analysis from the Economic and Social Research Council (ES/L01338X/1) to make a series of new trigger films and pursue two possible ways to streamline the discovery phase. | As part of a wider secondary analysis study to create new trigger films, we re-analysed interview transcripts on experiences of young people with depression and experiences of stroke. We then ran two workshops with people with relevant lived experience, working with extracts from the same materials after brief training. |
| Lucchese | Motivations of family advisors in engaging in research to improve a palliative approach to care for persons living with dementia: an interpretive descriptive study | 2024 | Canada | To explore PPI engagement in health research with family carers to understand further their interest in being involved in the SCG within the FCDS intervention | Semi-structured interviews were conducted by phone or videoconferencing and were recorded, transcribed, and analyzed using thematic analysis. |
| Luna Puerta | Perspectives on public involvement in health research from Singapore: The potential of a supported group model of involvement | 2020 | Singapore | 1. To investigate Singaporean public perspectives around the rationale, role and scope for being involved in health research  2.To identify the potential, challenges, facilitators and strategies for implementing PPI in Singapore. | Semi-structured qualitative interviews with members of the public, analysed using thematic framework analysis. |
| Malm | A balance between putting on the researcher's hat and being a fellow human being: a researcher perspective on informal carer involvement in health and social care research | 2022 | Sweden | To explore researchers’ views of involving informal carers in health and social care research. | Eleven individual in-depth interviews with researchers in the fields of social work, caring science, health science and medical science constituted the dataset of this qualitative study, inspired by discourse psychology. |
| Manikandan | Public and Patient Involvement in Doctoral Research During the COVID-19 Pandemic: Reflections on the Process, Challenges, Impact and Experiences From the Perspectives of Adults With Cerebral Palsy and the Doctoral Researcher | 2022 | Ireland | To provide an overview of how adults with CP were involved in a doctoral research process during the pandemic. | The data for this paper is a combination of reflection notes, email exchanges, meeting minutes and informal discussions with the PPI team on their experiences of being involved in the PPI process. |
| Mann | Reporting and appraising the context, process and impact of PPI on contributors, researchers and the trial during a randomised controlled trial - the 3D study | 2018 | United Kingdom | Using contributor and researcher perspectives and drawing on published guidelines for reporting PPI, we aimed to reflect on our experience and contribute evidence relevant to two important questions: What difference does PPI make? And What’s the best way to do it? | Fourteen people living with multiple long-term conditions (multimorbidity) were PPI contributors to a randomised controlled trial to improve care for people with multimorbidity. |
| Merker | Evaluating the Impacts of Patient Engagement on Health Services Research Teams: Lessons from the Veteran Consulting Network | 2022 | USA | To explore the impacts of engaging patients as consultants to research studies by examining the experiences, impacts, and lessons learned from a program facilitating patient engagement at a Veterans Health Administration research centre. | We conducted qualitative, semi-structured interviews with participants in the local Veteran Consulting Network to qualitatively explore these outcomes. |
| Meudell | "Hitting the spot": Developing individuals with lived-experience of health and social care as facilitators to deliver a course to enhance public involvement in research - a Welsh perspective | 2017 | United Kingdom | A project was initiated to develop members of the public as facilitators to deliver a public involvement in research course. | Three trainee facilitators were recruited to deliver a public involvement in research training course, the Building Research Partnerships course [5], as it was known then, originally developed for and by Macmillan Cancer Support. |
| Milley | Long-term consumer involvement in cancer research: Working towards partnership | 2021 | Australia | To identify barriers and facilitators to meaningful long-term consumer involvement in research. | Six semi-structured interviews were conducted with members of the Primary Care Collaborative Cancer Clinical Trials Group (PC4) Community Advisory Group CAG and included the review of 40 supporting documents. |
| Mitchell | An evaluation of the experiences of young people in Patient and Public Involvement for palliative care research | 2021 | United Kingdom | To evaluate the experiences of young people of Patient and Public Involvement for a paediatric palliative care research study. | Anonymous written feedback was collected from group members about their experiences of Patient and Public Involvement in a paediatric palliative care research study. An inductive thematic analysis of the feedback was conducted using NVivo. |
| Mitchell | Value and learning from carer involvement in a cluster randomised controlled trial and process evaluation - Organising Support for Carers of Stroke Survivors (OSCARSS) | 2020 | United Kingdom | This paper aims to describe PCPI in the OSCARSS study, a pragmatic-cluster randomised controlled trial with an embedded economic and process evaluation. | A carer research user group (RUG) co-developed OSCARSS to evaluate how to best deliver support to caregivers of stroke survivors. The PCPI activity involved regular meetings and preparatory work, from the initial conceptualisation of the study through to dissemination. Written reports, structured group discussions and individual interviews were carried out with the RUG and researchers to capture the added value and learning. |
| Mockford | A SHARED study-the benefits and costs of setting up a health research study involving lay co-researchers and how we overcame the challenges | 2016 | United Kingdom | The aim of this paper is to describe the benefits, challenges and costs involved in setting up the research study with lay members as part of the research team. | This began with a discussion of an initial research idea with a lay group of carers and people living with dementia. Once funded, approval was sought from the Research Ethics Committee and NHS Trusts to conduct the research including the active involvement of lay co-researchers. |
| Musson | Exploring patient and public involvement in motor neuron disease research | 2019 | United Kingdom | Our aim was to explore the experiences of those who participate in, organise and work with the SMNDRAG - Sheffield Motor Neurone Disorders Research Advisory Group. | We conducted 13 semi-structured interviews: ten with members of the SMNDRAG and three with researchers who have worked with the group. |
| Nissen | Patient involvement in the development of a psychosocial cancer rehabilitation intervention: evaluation of a shared working group with patients and researchers | 2018 | Denmark | The aim of this paper is to present our experiences from a shared working group (SWG) with patient representatives and researchers. | Data material for the present evaluation study was collected from meeting documents, transcriptions of interviews with two patient representatives and three researchers from the shared working group, and the primary investigator’s field notes. |
| Novak-Pavlic | Patients and Families as Partners in Patient-Oriented Research: How Should They Be Compensated | 2023 | Canada | To address the challenges and alternatives that go beyond payments only, with a particular focus on how compensation is addressed at different stages in the research process. | We present some of the resources that might help teams to navigate conversations about compensation with their patient and family partners and report how existing resources can be leveraged to compensate patient and family partners fairly and appropriately. |
| Oliver | Living with young onset dementia and actively shaping dementia research: The Angela Project | 2020 | United Kingdom | Keith shares his experience of receiving a diagnosis of dementia and how he has since been involved in research projects, highlighting the benefits of PPI for both the person living with dementia, as well as for the research group. | Keith shares his experience of receiving a diagnosis of dementia and how he has since been involved in research projects, highlighting the benefits of PPI for both the person living with dementia, as well as for the research group. |
| Patterson | Activity and views of service users involved in mental health research: UK survey | 2014 | United Kingdom | To describe activities, roles and experiences of service users involved in mental health research. | National cross-sectional online questionnaire survey, using snowball sampling. |
| Paul | Involving the public in mental health and learning disability research: Can we, should we, do we? | 2017 | United Kingdom | This study explored the attitude of researchers working in mental health and learning disability services in the UK towards PPI in health research. | Using a qualitative methodology, semi-structured interviews were conducted with a purposive sample of eight researchers. |
| Pritchard | Enhancing community engagement, public involvement, and social capital through researchers' participation in community dance projects: unexpected outcomes in underserved communities | 2024 | United Kingdom | To explore the impact of the Dance and Health project on the social capital of participants and provide key learnings on how to engage and build partnerships with people from underserved groups in health research contexts. | Qualitative interviews and focus groups were completed which explored participant and dance tutor experiences in community venues. |
| Repisti | Experiences of patients advising on mental health research: Qualitative study in South-East European countries | 2022 | Bosnia and Herzegovina, Kosovo, Montenegro, North Macedonia and Serbia | To explore experiences of patients participating in newly set up lived experience advisory panels (LEAPs) within a European Commission funded, large-scale, multi-country mental health research project that focused on improving treatment of individuals with psychosis. | Twenty-one mental health patients were individually interviewed across five countries. |
| Richmond | Creating positive experiences of involvement in mental health research | 2023 | United Kingdom | To explore what the differences have been and why, and to identify barriers and enablers to involvement that can promote the wellbeing of everyone involved. | A retrospective reflective approach was undertaken collaboratively by four people bringing lived experiences of mental health difficulties to a study adapting a social network intervention for mental health services. |
| Rossvoll | Patient and public involvement in health research from researchers' perspective | 2023 | Norway | To explore PPI in health research from the perspective of OT-trained researchers. | Semi-structured individual interviews were conducted online with nine Norwegian researchers. |
| Rossvoll | What motivates public collaborators to become and stay involved in health research? | 2024 | Norway | to investigate why people became public collaborators in health research and what helped sustain their commitment to staying involved. | In this qualitative study, we interviewed 11 people with experience from involvement, based on their experiential knowledge as patients or next-of-kin, in health research |
| Saini | The value of involving patients and public in health services research and evaluation: a qualitative study | 2021 | United Kingdom | To identify how public advisors were included, the impact of their involvement, and how change occurred within the organisations following their involvement. | A qualitative approach using focus group discussions was adopted to explore the experiences of two cohorts of participants involved in PPP project teams. |
| Schandl | Patient and public involvement in oesophageal cancer survivorship research | 2022 | Sweden | To describe and evaluate the development of PPI in oesophageal cancer survivorship research in Sweden by the use of a framework to support the process. | Insights, benefits, and challenges of the process were described and discussed among the collaborators. |
| Schilling | Patient involvement in clinical trials: motivation and expectations differ between patients and researchers involved in a trial on urinary tract infections | 2019a | Germany | Our aim was to study the motivation and expectations of patients and researchers towards patient and public involvement (PPI). | Prior to the first board meeting, we conducted telephone interviews with all researchers and patients regarding their motivation for involvement in the patient board and their expectations. |
| Schilling | Patients' and researchers' experiences with a patient board for a clinical trial on urinary tract infections | 2019b | Germany | To analyze the experience of patients and researchers with PPI in a clinical trial in Germany, so we could learn more about potential challenges and how they could be addressed. | We established a patient board for a randomized controlled trial on urinary tract infections, where patients and researchers regularly met to discuss relevant aspects of the trial. Minutes were taken for each meeting and the moderator also noted her observations in a postscript. After four meetings, we conducted two focus groups, one each with the patients and researchers. |
| Simpson | Adding SUGAR: service user and carer collaboration in mental health nursing research | 2014 | United Kingdom | This paper will describe the background to SUGAR and how and why it was established; how the group operates; some of the achievements to date including researcher reflections; and case studies of how this collaboration influences our research. | National cross-sectional online questionnaire survey, using snowball sampling. Descriptive statistics and framework analysis undertaken collaboratively with a service user reference group. |
| Slade | Developing involvement during a programme of recovery research | 2016 | United Kingdom | To consider the process of working with a Lived Experience Advisory Group (LEAP) and its outcomes in REFOCUS, a large 5 year programme of recovery research. | Narrative reflections on the experience of working with LEAP were collected from five members and the chair of LEAP, two REFOCUS researchers and the principal investigator. |
| Smith | Mixed methods study exploring parent engagement in child health research in British Columbia | 2019 | Canada | The objective of this study was to explore parent perspectives of and interest in an interactive knowledge translation platform called Child-Sized KT that proposes to catalyse the collaboration of patients, families, practitioners and researchers in patient-oriented research at British Columbia Children’s Hospital (BCCH). | Over 500 parents across BC completed an online survey, including a subsample of 102 parents who had accessed care at BCCH within the past 2 years. Following the online survey, two focus groups were held with parents in the Vancouver area to explore themes emerging from the survey. |
| Smith | Patient and public involvement in preclinical and medical research: Evaluation of an established programme in a Discovery-Based Medical Research Institute | 2024 | Australia | To evaluate an established patient and public involvement programme operating in a major Australian Discovery-Based Medical Research Institute (DBMRI) to inform programme development and the wider field. | Consumer/researcher co-developed online surveys and semi structured virtual interviews. |
| Stage | Exploring the hidden demands for patients’ resources: How health researchers risk excluding patients from patient and public involvement in health research during recruitment. | 2023 | Denmark | To explain challenges connected to recruitment, as health researchers seek to involve citizens in health research. | Through interviews with nurses and doctors who conduct research, I describe how and why different patients are recruited into research in the Danish healthcare system. |
| Stewart | Evaluating participant experiences of Community Panels to scrutinise policy modelling for health inequalities: the SIPHER Consortium | 2024 | United Kingdom | To describe our experiences involving members of the public in the SIPHER Consortium, a policy modelling programme with researchers and policymakers working together over five years to try to address health inequalities. | We focus on evaluating people’s experiences as part of Community Panels for SIPHER. |
| Thompson | Exploring the impact of patient and public involvement in a cancer research setting | 2014 | United Kingdom | Drawing on qualitative data with people involved in the National Cancer Research Network in the United Kingdom, we report on what motivated participants to get involved and their experiences of involvement in this setting. | Written reflective narratives of service user and carer experiences of SUGAR were analysed using constant comparative methods by the members. |
| Tremblay | Engaging indigenous patient partners in patient-oriented research: lessons from a one-year initiative | 2020 | Canada | The 'Indigenous patient partners platform project' was a small-scale initiative aimed to address the issue of the underrepresentation of Indigenous people in patient-oriented research by recruiting, orienting and supporting Indigenous patient partners in Quebec (Canada). | The evaluation of this initiative used a case study design hinging on documentary analysis and committee member interviews. Project documents (n= 29) included agendas and meeting minutes, support documents from the orientation workshop and workshop evaluations, and tools the committee developed as part of the project. |
| Tripp | Evaluating the impacts of patient engagement on a national health research network: results of a case study of the Chronic Pain Network | 2023 | Canada | The objective of this exploratory evaluation case study was to understand the impacts of patient engagement on the CPN | Interviews were conducted with CPN members, including patient partners, leadership, funded researchers and committee co-chairs, at three discrete time points to trace the evolution of the patient engagement program within the Network. |
| Vat | Giving patients a voice: a participatory evaluation of patient engagement in Newfoundland and Labrador Health Research | 2020 | Canada | In this pilot evaluation study, we aimed to 1) evaluate patient engagement in health research projects in Newfoundland and Labrador, Canada, and 2) learn more about how to best monitor and evaluate patient engagement. | A formative evaluation of patient engagement in health research projects. Participants completed an online survey. |
| Warner | I felt like a human being'-An exploratory, multi-method study of refugee involvement in the development of mental health intervention research | 2021 | Sweden | To describe the group dynamic characteristics and immediate impact of PPI from the user representatives' perspective in a case study of refugee involvement in the development of mental health intervention research. To pilot and methodologically appraise the Active Involvement of Users in Research Observation Schedule and Questionnaire. | The Active Involvement of Users in Research Observation Schedule and Questionnaire were administered together with a focus group discussion (35 minutes) after 8 hour research meeting. |
| Wilson | ReseArch with Patient and Public invOlvement: a realisT evaluation: the RAPPORT study | 2015 | United Kingdom | To determine the types of PPI in funded research, describe key processes, analyse the contextual and temporal dynamics of PPI and explore the experience of PPI in research for all those involved. | The first two stages comprised a scoping exercise and online survey to chief investigators to assess current PPI activity. The third stage consisted of case studies tracked over 18 months through interviews and document analysis. |

**Additional File 7: Payment for Involvement**

| Author | Rate paid | Activities paid for | Challenges |
| --- | --- | --- | --- |
| Barn 2022 | Rate not stated | Travel expenses, members offered gift cards as  compensation for their time and contribution. |  |
| Bayliss 2017 | £75 per meeting | Meeting | Observations, analysis and contributions to the writing of this paper were on a voluntary basis |
| Beland 2022 | Rate not stated | Travel expenses, payment for time | Some patient partners mentioned being compensated for attending meetings, but not necessarily for the time spent to prepare for them |
| Belisle-Pipon 2021 | $35 per online research activity (proposed rate) | Per online research activity | Disagreement with Institutional Review Board about who should be paid, budget restrictions resulted in researchers not compensating any of the advisory board members |
| Brighton 2018 | Rate not stated | Travel expenses, refreshments | Number of focus groups limited by funding |
| Chudyk 2024 | Patient partner participants were offered a $250 honorarium and academic researcher participants were offered a $100 honorarium (electronic gift card or cheque) (1.5 - 3h workshop) | Virtual workshop attendance |  |
| Coupe 2019 | Contributor: £75 half day work via expense forms; £20 per hour cash/voucher | Time spent for focus group, reviewing a booklet, giving feedback |  |
| Dawes 2024 | Rate not stated | Travel expenses, ‘thank you vouchers’ and lunch costs |  |
| Dawson 2020 | £20 per hour | Meetings, reimbursed for travel, carer allowance, childcare |  |
| De Wit 2013, 2017 | Rate not stated | Travel and expensed to international meetings |  |
| Di Lorito 2020 | Rate not stated | Time spent working on the process evaluation in any of the stages of the study | Not originally costed in proposal/ protocol |
| Evans 2014 | £14.02 - 19.77 per hour via claims forms | Meetings, work outside meetings (e-mails, collecting and analysing data, and writing) |  |
| Evans 2020 | Salaried researchers |  |  |
| Evans 2022 | Rate not stated | Paid honoraria and travel |  |
| Fleming 2021 | $50 per meeting |  |  |
| Foster 2021 | In line with NIHR guidance, in cash/bank transfer, in ‘high street’ shopping vouchers, or in kind, for instance, one member chose to receive books | Meetings, preparation, travel, accommodation, incidentals | Over the course of the project, the university’s process for paying people for such activities changed and members were subsequently required to submit an invoice for each payment, which HC and RF supported. |
| Giel, 2023 | None provided | Travel expenses only |  |
| Hoven 2020 | Rate not stated | Travel expenses, accommodation, project meetings, assignment time |  |
| Howells 2023 | £50 per meeting | Meeting attendance (1.5-2 hours) |  |
| Jorgenson 2018 | Agreed no payment |  | Logistics of payments frustrating |
| Lindenmeyer 2007 | £75 per month | Agreed travel expenses only |  |
| Locock 2019 | Rate not stated | 5 hour workshop, including refreshments, lunch, social time. Travel expenses paid |  |
| Luna Peurta 2020 | 15SDG voucher |  |  |
| Mann 2018 | £12 (vouchers) | Meeting time, preparation, work between meetings, travel (cash), lunch provided |  |
| Merker 2022 | $25 per hour | Meeting attendance |  |
| Mitchell 2010 | £10 (vouchers) | Preparation, meeting time, out of pocket expenses |  |
| Mockford 2016 | £18-20 | Time and travel expenses, care worker, formal training |  |
| Schilling 2019 | €50 per meeting | Patient board meeting and travel |  |
| Simpson 2014 | Rate not stated; Honorary university contract | Meetings and additional input |  |
| Slade 2016 | £150 per meeting plus travel. (5 hours with one hour lunch break) | Meeting and travel expenses |  |
| Tremblay 2020 | Rate not stated | Project meetings and evaluation | Writing time not budgeted for |
| Vat 2020 | Rate not stated |  | No suitable payment system |
| Warner 2021 | Hourly, rate not stated |  |  |
